# Neurogenic Bladder

**DOI:** 10.1155/2012/816274

**Published:** 2012-02-08

**Authors:** Peter T. Dorsher, Peter M. McIntosh

**Affiliations:** Department of Physical Medicine and Rehabilitation, Mayo College of Medicine, 4500 San Pablo Road, Jacksonville, FL 32224, USA

## Abstract

Congenital anomalies such as meningomyelocele and diseases/damage of the central, peripheral, or autonomic nervous systems may produce neurogenic bladder dysfunction, which untreated can result in progressive renal damage, adverse physical effects including decubiti and urinary tract infections, and psychological and social sequelae related to urinary incontinence. A comprehensive bladder-retraining program that incorporates appropriate education, training, medication, and surgical interventions can mitigate the adverse consequences of neurogenic bladder dysfunction and improve both quantity and quality of life. The goals of bladder retraining for neurogenic bladder dysfunction are prevention of urinary incontinence, urinary tract infections, detrusor overdistension, and progressive upper urinary tract damage due to chronic, excessive detrusor pressures. Understanding the physiology and pathophysiology of micturition is essential to select appropriate pharmacologic and surgical interventions to achieve these goals. Future perspectives on potential pharmacological, surgical, and regenerative medicine options for treating neurogenic bladder dysfunction are also presented.

## 1. Background

Normal micturition involves proper function of both the bladder and urethra. A detrusor of normal compliance and a physiologically competent urethral sphincter are both necessary to maintain urinary continence. Any increase in abdominal pressure, which inherently produces an increase in bladder pressure, is normally counteracted by an even greater increase in urethral pressure.

Normal micturition involves passive, low pressure filling of the bladder during the urine storage phase while voiding requires coordination of detrusor contraction with internal and external urinary sphincter relaxation. This micturition process is controlled by the central nervous system, which coordinates the sympathetic and parasympathetic nervous system activation with the somatic nervous system to ensure normal micturition with urinary continence [1].

Voiding dysfunction can result from any mechanical or physiologic defects in the micturition system that result in the inability of the urinary sphincter to appropriately increase (or decrease) its pressure in response to increased bladder pressure. Damage or diseases of the central, peripheral, and autonomic nervous systems may result in neurogenic bladder dysfunction.

Neurogenic bladder dysfunction may complicate a variety of neurologic conditions. In the United States, neurogenic bladder affects 40–90% of persons with multiple sclerosis, 37–72% of those with Parkinsonism, and 15% of those with stroke [2]. Detrusor hyperreflexia is seen in 50–90% of persons with multiple sclerosis, while another 20–30% have detrusor areflexia. There are more than 200,000 persons with spinal cord injuries, and 70–84% of these individuals have at least some degree of bladder dysfunction [3]. Bladder dysfunction is also common in spina bifida, which affects approximately 1 per 1000 live births. Vesicoureteral reflux may occur in up to 40% of children with spina bifida by age 5, and up to 61% of young adults with spina bifida experience urinary incontinence [4]. Less common causes of neurogenic bladder include diabetes mellitus with autonomic neuropathy, pelvic surgery sequelae, and cauda equina syndrome due to lumbar spine pathology.

Manack et al. [3] examined insurance and pharmacy claims of nearly 60,000 patients with neurogenic bladder over a 4-year period and found a 29–36% rate of lower urinary tract infections, 9–14% rate of urinary retention, and a 6–11% rate of urinary tract obstructions. Upper urinary tract infections were noted in 1.4–2.2% of the neurogenic bladder cohort, and serious systemic illnesses were also diagnosed in this group including septicemia in 2.6–4.7% and acute renal failure in 0.8–2.2%. Neurogenic bladder patients averaged 16 office and 0.5 emergency room visits per year, approximately a third of them leading to hospitalization.

Neurogenic bladder with detrusor overactivity may cause incontinence, which not only leads to embarrassment, depression and social isolation but also may lead to skin decubiti, urethral erosions, and upper urinary tract damage [5].

## 2. Anatomy and Physiology of the Bladder

The basic neuroanatomy and neurophysiology of the upper and lower urinary tracts should be understood before considering bladder management issues. Normal voluntary micturition includes bladder filling, storage, and emptying [1]. The kidneys receive nearly 25% of the cardiac output, filtering 180 L per day though only approximately 1 L/day is excreted as urine. This filtrate is transported through the ureters to the bladder. The ureters, which are approximately 25–30 cm in length, pass obliquely through bladder wall at the ureterovesicular junction to form a one-way valve that serves to prevent retrograde reflux of urine to the kidneys during bladder filling and emptying stages. This one-way valve mechanism remains competent only as long as the oblique course of the ureters is maintained thru the bladder wall. The bladder stores urine in a low pressure system with a normal capacity of 400–500 cc. Anatomically, the bladder is divided into the detrusor (aka as “body” or “dome” of the bladder), which consists of smooth muscle, and the base, which includes the trigone and bladder neck that are intimately connected to the pelvic floor. The bladder outlet has two urethral sphincters, the internal (smooth muscle) sphincter in the bladder neck and proximal urethra and the external (striated muscle) sphincter of the membranous urethra. Females have a less complex urinary sphincter mechanism that surrounds a shorter urethra.

**Figure 1:**
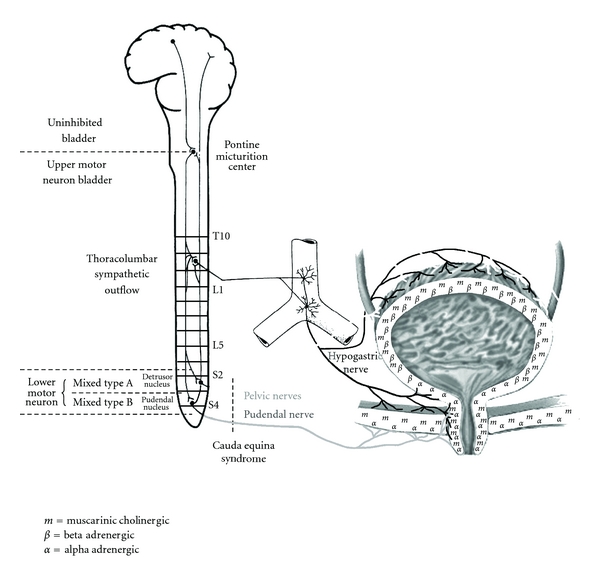
Anatomy and physiology of micturition with potential injury sites to urologic system (*m*: muscarinic receptor, *α*: alpha-adrenergic receptor, *β*: beta-adrenergic receptor).

Regulation of micturition involves cortical, subcortical, brainstem, spinal cord, and bladder mechanisms (see Figure 1). Cortical control areas in the frontal and cingulate gyri as well as subcortical areas provide inhibitory influence on micturition at the level of the pons and excitatory influence on the external urinary sphincter. This allows voluntary control of micturition so that normally bladder evacuation can be delayed until an appropriate time and place to void are chosen.

The pontine micturition center (PMC, also known as Barrington's nucleus or M-region) is essential for the coordination of micturition. This is accomplished by the PMC modulating the opposing effects of the parasympathetic and sympathetic nervous systems on the lower urinary tract. In the bladder emptying stage, the PMC sends excitatory influence to the sacral spinal cord that produces detrusor contraction while simultaneously sending inhibitory influence to the thoracolumbar cord that produces internal urinary sphincter relaxation. The overall effect is to allow evacuation of the bladder contents. Conversely, during bladder storage phase, PMC inhibition causes suppression of sacral spinal cord that produces detrusor relaxation while simultaneously sending excitatory influence to the thoracolumbar cord that produces internal urethral sphincter contraction. The overall effect is to allow filling/storage of urine in the bladder. More details of these mechanisms will be discussed further below.

Ascending sensory information on the state of bladder filling is believed to reach the periaqueductal gray (PAG) matter where it is then relayed via the hypothalamus and thalamus to the anterior cingulate cortex, insula, and prefrontal cortex. These brain regions inhibit the PAG, which itself has excitatory input to the PMC. The hypothalamus has excitatory influence on the PAG. When the conscious decision to void occurs, the prefrontal cortex inhibition of the PAG is interrupted while simultaneously the hypothalamus stimulates the PAG. The overall result is excitation of the PMC which produces voiding.

Spinal neurons involved in the regulation of micturition are located in the dorsal commissure, superficial dorsal horn, and parasympathetic nucleus. Interneurons send rostral projections but also serve to regulate spinal segmental reflexes. Glutamate serves as the excitatory transmitter at the spinal level while glycine and *γ*-aminobutyric acid (GABA) are the inhibitory neurotransmitters.

Three mixed sensory and motor nerves (hypogastric, pelvic, and pudendal nerves) innervate the lower urinary tract. The hypogastric nerve carries sympathetic autonomic nervous system innervation, the pelvic nerve carries the parasympathetic autonomic nervous system innervation, and the pudendal nerve carries the somatic nervous system innervation to the lower urinary tract.

As shown in the Figure 1, sympathetic nervous system innervation to the lower urinary tract arises from the T11-L2 cord level to synapse in the inferior mesenteric and hypogastric plexuses before continuing via the hypogastric nerves to *α*-adrenergic receptors in the bladder neck and proximal urethra as well as *β*-adrenergic receptors in the bladder fundus. Sympathetic nerve fibers also innervate parasympathetic ganglia in the detrusor wall and have an inhibitory effect on those ganglia. Activation of the thoracolumbar sympathetic outflow produces norepinephrine release in the lower urinary tract that results in detrusor relaxation and bladder neck (internal sphincter) contraction.

Parasympathetic nervous system innervation to the lower urinary tract arises from the detrusor nucleus at the S2–S4 cord level (see Figure 1) which passes through the pelvic nerves to cholinergic parasympathetic neurons in ganglia in the detrusor. Acetylcholine released by activation of these neurons produces detrusor contraction through M2 and M3 muscarinic receptor activation, though M1 receptors are also present in prejunctional neuronal terminals. Parasympathetic innervation in the proximal urethra causes nitric oxide to be released there which produces urethral smooth muscle relaxation. Activation of the sacral parasympathetic outflow produces acetylcholine and nitric oxide in the lower urinary tract that results in detrusor contraction and relaxation of the proximal urethra.

Somatic nervous system innervation to the external urethral sphincter arises from the pudendal (Onuf's) nucleus at the S2–S4 cord level which then passes through the pudendal nerve to the sphincter striated muscle. Supraspinal centers, which normally are under voluntary control, produce excitatory influence on the pudendal nucleus during the bladder filling stage to produce external urethral sphincter and pelvic floor contraction to help maintain continence, while during bladder voiding stage this descending influence is inhibited to produce urethral and pelvic floor relaxation that facilitates bladder emptying.

Afferent information on the state of bladder filling is transmitted from sensory fibers in dense suburothelial and muscular plexuses. Some sensory fibers may extend through the urothelium into the bladder cavity to transduce both physical and chemical stimuli. The vast majority of these sensory afferents are small myelinated A*δ* fibers and unmyelinated C fibers. The A*δ*  fibers respond to bladder wall distention and trigger micturition, while C fibers respond to painful stimuli. The majority of the bladder afferent fibers run in the pelvic nerves to the sacral dorsal root ganglia, and after transduction of these signals in the dorsal horn of the spinal cord, this sensory information is transmitted rostrally to the PAG region, as previously described.

During the bladder filling stage, supraspinal centers produce inhibition of the pontine micturition center, which results in enhancement of thoracolumbar sympathetic outflow with simultaneous suppression of sacral parasympathetic outflow to the lower urinary tract. These supraspinal centers also produce excitatory outflow through the pudendal nerve to produce external urethral sphincter contraction. The overall effect in normal bladder physiology is detrusor smooth muscle relaxation, bladder neck smooth muscle contraction, and external urinary sphincter skeletal muscle contraction that allow low pressure storage of urine in the bladder without leakage.

During the bladder emptying phase, the supraspinal centers' inhibitory outflow to the pontine micturition center is suppressed, resulting in reduction of thoracic sympathetic outflow with simultaneous enhancement of sacral parasympathetic outflow to the lower urinary tract. The supraspinal centers' excitatory outflow through the pudendal nerve is suppressed producing external urethral sphincter relaxation. The overall effect in normal bladder physiology is detrusor smooth muscle contraction, bladder neck smooth muscle relaxation, and external urinary sphincter skeletal muscle relaxation that allow evacuation of urine stored in the bladder.

## 3. Pathophysiology of Neurogenic Bladder

Many classifications have been used to group neurogenic bladder dysfunction. Each has their merits and clinical utility. These classifications may be based on urodynamic findings (e.g., Lapides [6], Krane, and Siroky [7]), neurourologic criteria (Hald and Bradley [8], Bors and Comarr [9]), or on bladder and urethral function (International Continence Society [10], Wein [11]).

A popular classification of neurogenic bladder dysfunction based on the location of the neurologic lesion can help guide pharmacologic and surgical therapies, with the voiding abnormalities seen clinically following from disruptions of the normal urinary physiology described above and shown in Figure 1. In this classification, neurogenic bladder arises from

1. lesions above the pontine micturition center (e.g., stroke or brain tumor) producing an *uninhibited bladder, *
2. lesions between the pontine micturition center and sacral spinal cord (e.g., traumatic spinal cord injury or multiple sclerosis involving cervicothoracic spinal cord) producing an *upper motor neuron bladder, *
3. sacral cord lesions that damage the detrusor nucleus but spare the pudendal nucleus producing a *mixed type A bladder, *
4. sacral cord lesions that spare the detrusor nucleus but damage the pudendal nucleus producing a *mixed type B bladder, *
5. *lower motor neuron bladder * from sacral cord or sacral nerve root injuries.

In uninhibited neurogenic bladder dysfunction, there is usually reduced awareness of bladder fullness and a low capacity bladder due to reduction of inhibition of the pontine micturition center (PMC) by cortical and subcortical structure damage. Urinary incontinence may occur with brain lesions occurring above the pontine micturition center, especially with bilateral lesions. Since the PMC is intact, the normal opposition of detrusor and internal/external sphincter tonus is maintained so there are no high bladder pressures developed that can lead to upper urinary tract damage.

Upper motor neuron neurogenic bladder dysfunction is characterized by detrusor-sphincter dyssynergia (DSD), wherein simultaneous detrusor and urinary sphincter contractions produce high pressures in the bladder (up to 80–90 cm H_2_O) leading to vesicoureteral reflux that can produce renal damage. The spinal cord damage renders the bladder and sphincters spastic, especially if lesions are above the T10 level (above the sympathetic autonomic nervous system innervation of the bladder). The bladder capacity is usually reduced due to the high detrusor tonus (neurogenic detrusor overactivity, or detrusor hyperreflexia). Animal studies suggest that activation of prejunctional M1 receptors facilitates acetylcholine release, so excessive release of this neurotransmitter due to upper motor neuron lesions may be a mechanism by which neurogenic detrusor overactivity occurs. As the bladder hypertonicity produces hypertrophy of the detrusor muscle, the normal oblique course of the ureter through the detrusor wall at the ureterovesicular junction is compromised to allow vesicoureteral reflux. If detrusor pressure exceeds internal/external urinary sphincter pressure in the proximal urethra, then incontinence may occur.

In the mixed type A neurogenic bladder (the more common of the mixed type bladders), detrusor nucleus damage renders the detrusor flaccid (also referred to as detrusor areflexia), while the intact pudendal nucleus is spastic producing a hypertonic external urinary sphincter. The bladder is large and has low pressure, so the spastic external sphincter produces urinary retention. The detrusor pressure is low so upper urinary tract damage from vesicoureteral reflux does not occur, and incontinence is uncommon.

The mixed type B neurogenic bladder is characterized by a flaccid external urinary sphincter due to the pudendal nucleus lesion while the bladder is spastic due to the disinhibited detrusor nucleus. Thus, the bladder capacity is low but vesicular pressures are usually not elevated since there is little outflow resistance. This leads to problems with incontinence, however.

In lower motor neuron neurogenic bladder, the sacral micturition centers or related peripheral nerves are damaged though the thoracic sympathetic nervous system outflow to the lower urinary tract is intact. The bladder capacity is large since detrusor tone is low (detrusor areflexia) and internal urinary sphincter innervation is intact. Despite the low detrusor pressure, overflow urinary incontinence and urinary tract infections are not uncommon.

Another type of bladder dysfunction was first described in nursing home residents, termed detrusor hyperactivity with impaired bladder contractility (DHIC), in which there is frequent but weak involuntary detrusor contractions causing incontinence despite incomplete bladder emptying [12] (Resnick). DHIC is associated with bladder trabeculation, slow bladder contraction velocity, and elevated urinary residual volume after voiding attempts.

## 4. Neurourological Evaluation

This evaluation is essential to assess lower urinary tract function.

A full patient history should be obtained including prior genitourinary conditions/surgeries, voiding history, voiding complaints (dysuria, recurrent infections, hesitancy, nocturia, incontinence, urgency, and/or frequency), and medications. Sedative/hypnotic, antidepressant, antipsychotic, antihistamine, anticholinergic, antispasmodic, opiate, alpha adrenergic agonists/antagonists, and calcium channel blocking medications may affect voiding function. Optimally, a patient urinary diary with voiding pattern, fluid intake, and voiding issues can help with the patient evaluation and formulation of treatment recommendations.

A physical examination focusing on pelvic anatomy and the neurologic system is essential. Neurological examination should include mental status, reflexes, strength, and sensation (including sacral dermatomes) to determine if there are neurologic conditions present that may contribute to the voiding dysfunction. Mechanical issues such as prostate enlargement or bladder prolapse can be found on urologic exam that may impact voiding function. Issues with cognition, hand strength and coordination, joint contractures, mobility, sexuality, social/medical support, and other factors may impact the type of bladder rehabilitation that is feasible for a given patient. For spinal cord injured patients, the motor level of spinal lesion, whether the injury is complete or incomplete, extremity tone, rectal sensation/tone, presence/absence of voluntary rectal tone, and bulbocavernosus reflex should be determined.

Laboratory evaluation of neurogenic bladder patients should include urinalysis, urine culture and sensitivity, serum BUN/creatinine, and creatinine clearance [13]. Post-void residual (PVR) urine volume involves transurethral catheterization to measure residual urine volume in the bladder immediately after voiding to determine the ability of the bladder to empty completely. PVR determination should always be performed after discontinuing Foley catheterization or before instituting intermittent catheterization as part of a bladder retraining program. The PVRs are necessary to prevent overdistension of the bladder and determine the frequency of catheterization that is needed to keep residual urinary volumes under approximately 400 cc. Abnormal residual volumes have been defined by volumes greater than 100 cc or greater than 20% of the voided volume, and residual urine volumes under 100 cc are associated with a reduced risk of development of bacterial cystitis [14]. Ultrasound is a noninvasive means of determining urine post-void residual volumes, especially if precise measurement is not required [15].

 Renal clearance tests including twenty-four-hour urine creatinine clearance and several isotope studies can be used to evaluate and sequentially follow renal function in neurogenic bladder patients. A commonly used test measures the glomerular filtration rate via a short renal clearance test using 125 I-iothalamate [16].

Urodynamic evaluation should be completed to assess urinary function, including urinary flowmetry, bladder cystometrogram/electromyogram (CMG/EMG), Valsalva leak point pressure (LPP) measurement, and urethral pressure profile (UPP). Urodynamic studies are the most definitive and objective means to determine abnormalities in the bladder and urethra in the filling/storage phase as well as voiding phase in neurogenic bladder dysfunction.

Urinary flow rate evaluation is a noninvasive way to quantify urinary flow, defined as the volume of urine voided per unit of time. Urine flow is dependent on the force of detrusor contraction as well as the urethral resistance. Normal urine flow curves are bell-shaped with rapid rise to peak flow, a short duration of peak flow, and then rapid falloff of urine flow. Urine flow rate patterns are not diagnostic, but high flow rates are often seen with neurogenic detrusor overactivity, and poor flow rates may reflect weak detrusor pressure and/or urinary outlet obstruction.

The bladder cystometrogram/electromyogram testing uses gas or liquid to fill the bladder to allow assessment of bladder volume, compliance, and sensation as well as documenting whether uninhibited bladder activity is present. Bladder pressures are monitored during filling and emptying by use of a transurethral catheter connected to a pressure transducer, and intra-abdominal pressure is often measured as well. During the examination, bladder sensations are determined including sensation of bladder filling (which normally occurs between 100–200 cc), first urge to void (which normally occurs between 300–400 cc), and strong urge to void (which normally occurs between 400–500 cc). Normal bladder capacity is 300–600 cc generally. Sphincter electromyography using surface or needle electrodes is performed simultaneously to determine if voiding is coordinated or not (i.e., whether detrusor sphincter dyssynergia is present).

Bladder leak point pressure is the maximum detrusor pressure measured during passive filling before urine leakage occurs. Sustained high detrusor pressures may occur in neurogenic bladders having poor compliance, and leak point pressures over 40 cm H_2_O places the upper urinary tract at increased risk for damage [17].

Urethral pressure profile (UPP) is a measure of the outflow resistance within the urethra. Several techniques have been described but most commonly it is measured by recording pressures along the length of the urethra during slow withdrawal of a water-filled urethral catheter connected to a pressure transducer. UPP is most commonly performed after cystometry, and thus comparing the peak urethral pressure to the pressures measured in the detrusor during cystometrogram can provide clinically beneficial information about bladder emptying potential. UPP has several clinical applications including the diagnosis of stress urinary incontinence, urethral instability, and urethral diverticula, but its utility for diagnosing and treating neurogenic bladder is less clear [18].

## 5. Neurogenic Bladder Management

Management of neurogenic bladder conditions requires patient education and may include interventions such as timed voiding, manual expression, medications, intermittent catheterization, indwelling urinary catheter, and bladder and/or urethral surgical procedures.

### 5.1. Nonpharmacologic Interventions

#### 5.1.1. Bladder Retraining and Fluid Schedule

The goals of management of neurogenic bladder are to (1) achieve/maintain continence to avoid the psychological and physical (e.g., skin maceration and decubiti) consequences of incontinence, (2) prevent development of a high pressure detrusor that can lead to upper urinary tract damage, (3) minimize risk of symptomatic urinary tract infections, and (4) prevent over-distension of the bladder.

 Initiation of a fluid schedule is an important initial intervention in the management of neurogenic bladder patients requiring intermittent catheterization as part of their neurogenic bladder management and may also be of help in patients with uninhibited neurogenic bladder dysfunction. A fluid schedule allows a predictable degree of bladder filling without overdistending the detrusor. Repetitive overdistension of the detrusor can lead to permanent muscle damage, producing a flaccid, large capacity bladder (i.e., a myogenic bladder). This is especially important if the neurogenic bladder dysfunction is potentially a temporary clinical situation (e.g., urinary retention following cauda equina syndrome from lumbar disc herniation).

The recommended fluid schedule is 400 cc with meals; 200 cc at 10 am, 2 pm, and 4 pm; and then only sips of fluid after the evening meal (1800 cc/day total). Accounting for insensible fluid loss with respiration and sweating, this fluid schedule keeps urine formation limited to about 1600 cc per day. If urinary catheterization is then performed on an every 6 hours schedule, the catheterized volumes will be approximately 400 cc each. This prevents bladder overdistension, and catheterization should be frequent enough to optimally keep bladder volumes to 400–500 cc. If neurogenic bladder management consists of an indwelling Foley catheter or suprapubic catheter, then fluid intake should be generous. The presence of the catheter in the bladder results in bacterial colonization there that produces a biofilm on the catheter tip [19]. Urease producing bacteria in the bladder elevate urine pH causing calcium and magnesium phosphate crystals to form in the urine which cause encrustation of the catheter and potentially obstruction [20]. Obstruction of the catheter is associated with an elevated risk of urinary tract infections as is low urinary output [21]. Increasing dietary citrate to at least 11 g per day in fruit juices to keep urine pH  > 8 in conjunction with increasing fluid intake to dilute urine calcium and magnesium concentrations [22] can prevent catheter encrustation, especially if total daily fluids are at least 3 liters per day [20].

 Individualized patient education regarding management of their neurogenic bladder via specialized nursing is important for achieving successful neurogenic bladder management. Nurses can monitor patient compliance with fluid schedule and teach patients how to perform timed voids as well as manual techniques including self-catheterization, Crede technique (manual pressure over suprapubic region to increase vesicular pressure to promote bladder emptying), tapping (over suprapubic area to trigger detrusor contraction in hyperreflexic bladders), and/or Valsalva maneuver (which increases vesicular pressure and may also trigger detrusor contraction).

The use of incontinence pads or external urinary collection devices such as condom (Texas) catheters as the sole interventions for long-term management of neurogenic bladder dysfunction is rarely appropriate. Absorbent incontinence pad use can lead to skin maceration and breakdown and increase the risk of urinary tract infections about 4 fold [23]. This may be mitigated by frequent change of pads. Condom catheters are appropriate for incontinent males without urinary retention who have significant functional impairments [24]. For example, a male with an uninhibited bladder and severe hemiparesis following a stroke may be an appropriate candidate for a condom catheter for management of his incontinence. Condom catheters are more comfortable and have a lower incidence of bacteriuria than indwelling urethral catheters [25]. Though skin breakdown commonly occurs, other complications such as urethral diverticuli and penile ischemia are uncommon [26].

Clean intermittent catheterization is the preferred method of bladder management for patients with neurogenic bladder dysfunction with partial or complete urinary retention. Regular bladder emptying reduces intravesical bladder pressure and improves blood circulation in the bladder wall, making the bladder mucous membrane more resistant to infectious bacteria [27]. Intermittent catheterization improves self-care and independence and reduces barriers to sexual intimacy compared to use of an indwelling catheter. If the patient with neurogenic bladder dysfunction has some ability to perform voluntary voiding, then voiding attempts are attempted every 3 hours while awake including just-before-intermittent catheterization attempts. If the voiding attempts enable the patient to empty the bladder sufficiently so that residual urine volume in the bladder is under 100 cc consistently, then the catheterization can be discontinued. Residual urine volumes under 100 cc are associated with a reduced risk of development of bacterial cystitis [14].

The tip of a catheter used for intermittent catheterization can be either straight or curved (referred to as Coudé-tipped). A closed intermittent catheterization system is available which is designed to reduce the risk of bacterial contamination of the bladder during insertion since the catheter never comes into direct contact with the inserter's hands. Medicare and Medicaid will cover these closed-catheter systems with introducer tips for persons requiring intermittent self-catheterization who are immune-compromised, nursing home residents, experiencing documented vesicoureteral reflux, pregnant spinal cord injured females with neurogenic bladder, and/or having two or more urinary tract infections per year with clean intermittent catheterization.

The most frequent complication of intermittent catheterization is urinary tract infection. Studies show that in patients with spinal cord injuries, the incidence of bacteria in the bladder is 1–3% per catheterization and that 1–4 episodes of bacteriuria occur per 100 days of intermittent catheterization. Wyndaele's review of the risks of intermittent catheterization indicates an 11% prevalence of asymptomatic urinary tract infections and 53% prevalence of symptomatic bacteriuria reported in various series [28]. The use of prophylactic antibiotics for intermittent catheterization is controversial, but use of low dose sulfamethoxazole or nitrofurantoin at night may reduce the risk of symptomatic bacterial cystitis without encouraging drug-resistant bacteria. Prostatitis may occur in up to a third of men performing intermittent catheterization, but epididymitis and urethritis are rare. Catheter trauma is common but generally does not produce lasting damage, though the prevalence of urethral strictures and false passages increases with duration of intermittent catheterization use. Rare complications of intermittent catheterization include formation of bladder stones, loss of catheter in the bladder, bladder perforation, bladder necrosis, and bladder cancer.

Indwelling Foley catheter placement for neurogenic bladder management is an option for uncontrollable incontinence or for urinary retention when intermittent catheterization is not practical. Estimates are that nearly 4 million Americans a year undergo indwelling urinary catheter placement, including up to a quarter of patients in acute care facilities and 5–10% of those in long-term care facilities for over a year [29, 30]. Duration of catheter use in home settings may be rather long, with a median of 3-4 years, and some individuals may use them for more than 20 years [31]. For example, myelomeningocele patients may have impaired motor and/or intellectual function that make-self-clean intermittent catheterization impossible so indwelling urinary catheters may allow them to maintain independence. One study noted 12% of their myelomeningocele cohort used indwelling catheter for long-term bladder management [32, 33]. In the setting of long-term indwelling urinary catheter placement, antimicrobial prophylaxis to prevent urinary tract infection should be avoided as it will result in development of drug-resistant bacterial organisms colonizing the bladder. Instead, as previously discussed, patients with indwelling Foley catheters should maintain high (>3 liters/day) oral fluid intake to keep the colonized bacteria flushed from the bladder and the catheter should be changed monthly. Other complications of indwelling catheters include bleeding, urethral or bladder injuries, bladder sediment/stones, catheter malfunction (including obstructions), bladder perforation, rectovesical fistula, and bladder cancer [29, 34–36]. For male patients, many experts recommend using the smallest diameter urinary catheter feasible and to tape it to the lower abdominal wall under low tension to minimize the risk of urethral erosions [37].

Suprapubic catheters have some advantages over urethral catheters including elimination of those urethral erosion risks. Suprapubic catheters also are usually easier to manage in terms of hygiene and catheter changes, have less chance of catheter kinking, are easily reversible, and may cause less interference with self-image and sexual intimacy than with use of Foley catheters when used for long-term bladder management. Significant surgical risks exist, however, for suprapubic catheter insertions. A retrospective study of 219 patients found a surgical complication rate of 10% (mortality rate 1.8%) with a 30-day complication rate of 19% [38]. These high complication rates likely reflect the underlying medical comorbidities in this patient population. Beyond expected elevated risks of urinary tract infections (21%) in long-term followup, a quarter of the cohort experienced catheter obstruction and less frequent complications included exit site infection/granulation/bleeding, bladder spasms, and catheter technical difficulties. Despite this, 71% of this cohort had prior unsatisfactory experiences with Foley catheters prior to opting for suprapubic catheter insertion, and nearly 90% preferred their suprapubic catheter over a urethral catheter. Bladder stones occur in up to 65% of individuals with suprapubic catheters compared to 30% of those performing intermittent catheterization [39]. There are rare reports of bladder cancer, though with less than half the risk associated with use of urethral catheters [36]. Suprapubic catheters should also be changed routinely, and some experts [40] recommend changing the catheter at the average time it takes for the catheter to obstruct for each patient (one to several weeks).

### 5.2. Pharmacologic Interventions

There are many different classes of drugs that are used to treat neurogenic bladder dysfunction as part of a comprehensive bladder management program.

#### 5.2.1. Tricyclic Antidepressant Drugs

Though initially developed for the treatment of depression, their significant adverse side effect profiles have made them second-line agents for that indication. Serious side effects of tricyclic antidepressants exist including sedation, orthostasis, and cardiac conduction blocks; these drugs should particularly be used with caution in older individuals having neurogenic bladder dysfunction. Tricyclics should not be used in setting of pregnancy. The anticholinergic side-effects of this class of drugs have been used to reduce bladder detrusor tone in neurogenic bladder dysfunction as an off-label (non-FDA approved indication) use.

Imipramine not only reduces bladder tone through its strong anticholinergic effects and antispasmodic properties but it also increases bladder internal sphincter tone through *α*-adrenergic agonist effect to further facilitate urine storage. Additionally, imipramine has a local anesthetic effect on bladder mucosa, which may further reduce bladder contractility through spinal reflex mechanisms. Imipramine thus may be useful to reduce urinary urgency and frequency in uninhibited bladder dysfunction. Amitriptyline has (relatively) less anticholinergic effects than imipramine, yet it is similarly effective in reducing detrusor tone. Amitriptyline has strong sedation properties and may also be helpful in the treatment of neuropathic pain conditions and insomnia.

#### 5.2.2. Anticholinergic (Antimuscarinic) Medications

This class of medication reduces reflex (involuntary) detrusor activity by blocking cholinergic transmission at muscarinic receptors and is the first-line option for treating neurogenic detrusor overactivity (NDO). Though available anticholinergic agents have similar efficacy, these drugs differ in terms of side effects and tolerability based on their muscarinic receptor selectivity and rate of drug distribution. Anticholinergic drugs that bind M1, M2, and M3 muscarinic receptors (nonselective) have more side effects than newer agents that are more selective for M2 and/or M3 receptors. Sustained release drug preparations also improve antimuscarinic drug tolerability by attenuating peak drug serum concentrations.

 Nonselective anticholinergic drugs that bind M1 receptors may produce memory and cognition impairments. Agents binding M2 receptors can produce QT interval prolongation causing tachycardia and arrhythmias. Anticholinergic drugs that bind M3 receptors may produce visual blurring, xerostomia, and constipation.

 Nonselective anticholinergic agents include oxybutynin, tolterodine, and trospium chloride. Oxybutynin, which was the first drug approved for treating bladder detrusor overactivity, is available in immediate and sustained release oral preparations as well as transdermal and topical gel formulations. Tolterodine also comes in immediate and sustained release oral preparations, and may produce less xerostomia and cognitive side effects than oxybutynin. Trospium also is available in immediate and sustained release oral preparations. Since trospium does not cross the blood brain barrier, cognitive side effects are diminished and it may have fewer drug-drug interactions compared to the other Nonselective anticholinergic agents.

 Anticholinergic drugs that are more selective for M2 and M3 receptors, have fewer cognitive side effects than Nonselective agents. Solifenacin and darifenacin are more selective for M3 receptors so these agents may be safer to use in individuals having heart disease but may tend to produce more constipation than Nonselective anticholinergic medications (consider avoiding in patients with preexisting constipation issues). Darifenacin dosing does not have to be adjusted for renal impairment. Fesoterodine is a competitive muscarinic antagonist that does not appear to cause QT prolongation or cognitive impairments. Imidafenacin is a selective anticholinergic drug currently undergoing clinical trials in the United States but available in Japan.

Trospium or fesoterodine may be better therapeutic options for patients with hepatic impairment, since they are not processed by the CYP3a4 system, while darifenacin should be avoided in patients with severe hepatic impairment. If bladder overactivity symptoms continue despite a maximum dose of an anticholinergic agent for a month without significant side effects, then addition of a tricyclic agent may provide a synergistic effect in reducing detrusor tone.

Manack et al. [3] found 63% of persons diagnosed with neurogenic bladder were taking one-anticholinergic medication and another 9% more than one. Over a one year period, 38% of neurogenic bladder discontinued their anticholinergic medication(s) and did not restart while over 80% of patients had interruption of their treatment with these medications. During the one year study period, 33.3% (15,415) of the neurogenic bladder cohort required hospitalization, 23.4% (10,819) had emergency room admissions, and 14.4% (6,646) resided in a nursing home. Over 21% of hospitalized patients in their cohort were diagnosed with lower urinary tract infections, and approximately 8% were hospitalized with sepsis/septicemia. Of the one-third of neurogenic patients that were hospitalized, 5.6% were diagnosed with urinary retention and 5.4% were diagnosed with obstructive uropathies.

 Propiverine is a drug with antimuscarinic and calcium antagonist properties that is available in Europe and Japan and is reported to have few adverse reactions (mainly dry mouth and blurred vision). Though mainly used for treating overactive bladder, it has been used in spinal cord injured patients where it increased bladder capacity over 100 cc and improved bladder compliance [41]. Residual urine volume, however, increased from 50 cc to 87 cc in the propiverine group.

#### 5.2.3. Cholinergic Agonists

Urecholine is a synthetic muscarinic agonist with no significant nicotinic effects. It can be used to promote detrusor contraction in mixed type A or lower motor neuron lesions. Urecholine is administered about one hour before meals and at bedtime as part of the bladder training program in which voiding attempts and often manual techniques (Valsalva or Crede) are performed before the scheduled intermittent catheterization attempts every 6 hours. As a cholinergic agonist, urecholine can produce side effects including hypotension, bradycardia, bronchoconstriction, nausea/vomiting, abdominal cramping, and diarrhea. It should be used with caution in persons with asthma, chronic obstructive pulmonary disease, hyperthyroidism, peptic ulcer disease, intestinal obstruction, urinary tract obstruction, coronary artery disease (especially if conduction blocks), or Parkinsonism.

#### 5.2.4. Alpha-2 Adrenergic Agonists

This class of medications can be used in neurogenic bladder dysfunction when the internal urinary sphincter is spastic, which occurs with detrusor sphincter dyssynergia in upper motor neuron bladder dysfunction. Alpha-2 adrenergic agonists cause presynaptic reduction of norepinephrine release at central and peripheral adrenergic terminals. Since the internal urinary sphincter has alpha-adrenergic innervation (see Figure 1), these agents can enhance bladder emptying by reducing bladder neck tone.

Alpha-2 adrenergic agonists are seldom, the sole medication used for treating neurogenic bladder dysfunction, since detrusor hyperreflexia must be addressed to prevent upper urinary tract damage. Even in the setting of complete or nearly complete bladder emptying, persistent elevation of detrusor pressures can produce progressive renal damage from hydronephrosis.

Clonidine and tizanidine are alpha-2 agonists that have been used for reducing bladder outflow resistance. These agents have also may be used clinically to reduce pain and skeletal muscle tone, which may potentially produce beneficial side-effects in treating neurogenic bladder dysfunction in spinal cord injured patients. Tizanidine is administered orally. Clonidine is available in oral and transdermal forms, and intrathecal administration of this drug has also been studied as a potential treatment modality for refractory neurogenic bladder dysfunction.

Common side effects of these drugs include fatigue, dizziness, lightheadedness, dry mouth, and constipation. Cardiac dysrhythmias and depression are uncommon but serious side effects.

#### 5.2.5. Alpha-1 Adrenergic Antagonists

Alpha-1 adrenergic antagonists such as dibenzyline, terazosin, tamsulosin, alfuzosin, and doxazosin produce peripheral postsynaptic blockade of alpha-adrenergic receptors in the bladder neck and proximal urethra to reduce urinary outflow resistance. Their vasodilating effect on arterial smooth muscle produces a reduction in blood pressure. Alpha-1 adrenergic antagonist drug side effect profiles are similar to those of the alpha-2 adrenergic agonists as outlined above.

#### 5.2.6. Benzodiazepines

Benzodiazepines such as diazepam are believed to exert their clinical effects by binding at a specific site on the GABA-A receptor to potentiate the effects of the inhibitory neurotransmitter GABA (gamma amino butyric acid). Benzodiazepines bind at spinal and supraspinal sites to reduce skeletal muscle tone, including the external urinary sphincter. Thus, diazepam has been used clinically to treat external sphincter spasticity from upper motor neuron or mixed type A neurogenic bladder dysfunction. The resulting reduction in bladder outflow resistance may allow more complete bladder emptying.

Side effects of benzodiazepines include sedation, delirium, respiratory depression, muscle weakness, constipation, and blurred vision. Benzodiazepenes can produce physical and psychological dependence as well.

#### 5.2.7. GABA-B Agonists

Baclofen is the most commonly used drug of this class clinically, and it is believed to exert its clinical effects through modulation of the GABA-B receptors at spinal and supraspinal levels to reduce skeletal muscle tone. Thus, like the benzodiazepines, baclofen can be used to treat external urinary sphincter spasticity in neurogenic bladder conditions. Baclofen has a clinical advantage over the benzodiazepines in this regard as it does not appear to cause any tendency towards psychological dependence.

 Baclofen can also be administered intrathecally for refractory spasticity conditions, especially where oral antispasticity agents produce excessive sedation or other intolerable side effects.

#### 5.2.8. Botulinum Toxin

Botulinum toxin blocks neuromuscular junction presynaptic vesicle fusion, which prevents acetylcholine release and thus blocks signal transmission across the neuromuscular junction [5]. It also acts on sensory afferent neurons and prevents the excitatory effects of nerve growth factor (NGF) on bladder function, which may contribute to its beneficial clinical effects in treating neurogenic bladder. Injection of botulinum toxin A into the bladder detrusor or external urinary sphincter produces a dose-dependent weakening of those muscles, which have a high concentration of cholinergic nerve endings (see Figure 1). Botulinum toxin A injections can produce long-lasting improvements in neurogenic detrusor overactivity, incontinence, and quality of life in spinal cord injury individuals. Since insurance coverage for botulinum toxin for these urologic indications is often limited, this mode of intervention should be reserved for individuals where oral and transdermal trials of medications have failed.

Dykstra et al. [42] first reported using botulinum toxin to successfully treat urinary symptoms by injection of external urinary sphincter of spinal cord injured patients having detrusor sphincter dyssynergia. Injection of botulinum toxin A into bladder detrusor muscle to treat neurogenic incontinence was described a decade later [43]. Botulinum toxin A injection in the detrusor and suburothelial regions of the bladder body can produce beneficial effects for up to nine months, though up to 70% of neurogenic detrusor overactivity patients may require catheterization after botulinum toxin injections due to urinary retention. Side effects of botulinum toxin injections are low, with a <1% risk of developing antibodies to the toxin [44] and about a 1% incidence of temporary distant muscle weakness [45]. Approximately 10% of patients do not obtain the desired clinical benefit from the botulinum toxin injections.

A large, retrospective trial of 200 neurogenic bladder patients receiving detrusor botulinum toxin injections demonstrated a >50% increase of mean bladder volume to first reflex detrusor contraction and maximum cystometric capacity, with a proportional decrease in maximum detrusor pressure. These benefits lasted more than 6 months and could be more cost effective than bladder augmentation surgery over a 5-year period if a 40% early and late complication rate of that surgery is assumed and the botulinum toxin effect lasted at least 5 months [46].

For instances in which neurogenic bladder dysfunction is refractory to these medications, several novel pharmacologic options are in development including intravenous, intravesicular, and intrathecal agents.

#### 5.2.9. Opioids

Agonists of the classical *μ*, *δ*, and *κ* opioid receptors such as morphine interfere with voiding, while antagonists of those receptors such as naloxone stimulate voiding. Intrathecal administration of the *δ*-receptor agonists inhibits detrusor contractions, while *κ*-receptor agonists administered intravenously reduce detrusor sphincter dyssynergia in experimental spinal cord injury animal studies [47].

Nociceptin (orphanin FQ) is an endogenous neuropeptide that binds to the nociception receptor (NOP). It inhibits the micturition reflex in neurogenic incontinence conditions but not in healthy persons, and intravesicular administration of this compound has been demonstrated to improve bladder function in spinal cord injured individuals [48].

#### 5.2.10. Vanilloids

Intravesical administration of vanilloid solutions, such as capsaicin or resiniferatoxin (RTX), reduces detrusor overactivity by selectively desensitizing the unmyelinated C-fiber sensory nerves that transmit urothelial pain and temperature sensation. At high concentrations, these drugs suppress C-fiber responses to stimulation for prolonged periods. Increased expression of transient receptor potential cation channel subfamily V member 1 (TRPV1) vanilloid receptors in urothelial cells and C-fibers occurs following spinal cord injury, and experimental evidence suggests that activation of these receptors may be involved in the resulting detrusor overactivity. Intravesicular administration of capsaicin or RTX then could serve to reduce detrusor hyperreflexia due to spinal cord lesions.

Clinical studies have confirmed that intravesicular vanilloid solutions do improve neurogenic detrusor overactivity associated with spinal cord injury or multiple sclerosis, including a double-blind, randomized controlled study demonstrating that intravesicular capsaicin significantly inhibited spinal cord-associated detrusor overactivity [49]. Intravesicular capsaicin acutely may, however, cause transient exacerbation of bladder symptoms which does not occur with intravesicular RTX. One small study of intravesicular RTX in neurogenic detrusor overactivity found resolution or improvement of urinary incontinence in 75% of patients with those urologic improvements sustained for at least a year in 58% [50]. RTX can also be administered intrathecally to treat detrusor overactivity.

 Clinical use of vanilloids has been hampered by their pungency and lack of stability of vanilloid solutions. Oral TRPV1 antagonists (e.g., GRC 6211) are being developed to avoid these issues, and in animal spinal cord injury studies these drugs reduce high intravesicular pressures and reflex bladder contractions [51].

#### 5.2.11. Nerve Growth Factor

Urinary nerve growth factor (NGF) has been demonstrated to be elevated in neurogenic detrusor overactivity (Jacobs). This neurotrophic factor induces changes in the excitability of bladder afferents, and sequestration of NGF improves bladder reflex activity in animal models [52]. Compounds are being developed to sequester NGF to treat neurogenic detrusor overactivity.

#### 5.2.12. Nitrous Oxide Agonists

Experimental evidence suggests nitric oxide synthase-staining neurons are present in high density in human urethral sphincters, with activation of this mechanism producing reduction in urethral pressure [53]. Theoretically, since the urethral sphincter has excellent blood supply, oral or sublingual nitrates (presently used to treat angina) could be used to reduce external sphincter pressure in patients with detrusor sphincter dyssynergia. Carbon monoxide also relaxes urethral smooth muscles, and carbon monoxide-synthesizing enzymes (hemeoxygenase-2) coexist with nitric oxide synthase enzymes in neurons supplying the external sphincter [54], so this physiologic mechanism offers another avenue for drug development in the future.

## 6. Surgical Interventions for Neurogenic Bladder

When nonpharmacologic and pharmacologic treatments fail to control neurogenic detrusor overactivity, then surgical options including neuromodulation are appropriate treatment options.

### 6.1. Procedures to Enhance Detrusor Storage

#### 6.1.1. Neuromodulation for Neurogenic Detrusor Overactivity

Unilateral or bilateral sacral nerve root stimulation technology used for overactive bladder treatment has also been applied to neurogenic detrusor overactivity. A recent systematic review and meta-analysis [55] of this topic reported a pooled success rate of 92% for sacral neuromodulation for neurogenic bladder and 24% complication rate, though a lack of randomized controlled trials was noted and mean followup of studies examined was only 26 months. The most frequent adverse events of sacral nerve root stimulation were lead migration and pain at the site of the implant. Removal of the lead or stimulator was the most frequent surgical intervention resulting from adverse events.

Long-term studies suggest the efficacy of sacral neuromodulation may disappear after 1–4 years. A long-term study of 12 adult neurogenic bladder patients undergoing sacral neuromodulation implantation by Hohenfellner et al. [56] found that one stimulator had to be removed in the immediate postoperative period, and stimulation was ineffective in another three patients. The other 8 patients had at least 50% improvement of lower urinary tract dysfunction for an average of 4.5 years, but then all but one sacral neuromodulation implant became ineffective after this time frame. A prospective randomized controlled study of 42 children (mostly with spina bifida) undergoing sacral neuromodulation for neurogenic bladder dysfunction management found better functional bladder capacity on urodynamics in the control group but higher leak point pressure in the implant cohort. Improved bladder compliance and functional bladder capacity were noted in the implant group for the first 9 months of followup, but not after 12 months [57]. 

Combining dorsal rhizotomy to reduce detrusor pressure and sacral root stimulation to drive micturition has been described to treat upper motor neuron bladder dysfunction in female paraplegics, though this combination of procedures can be used in male paraplegics and quadriplegics as well as with the caveat that reflex erections (if present) may be lost due to the rhizotomy [58].

Chronic pudendal nerve stimulation has been studied for urge incontinence due to neurogenic detrusor overactivity [54]. Though followup in the study of Spinelli et al. was only 6 months, the twelve individuals undergoing chronic pudendal nerve stimulation more than doubled their bladder capacity to over 330 cc with 56% reduction of maximum detrusor pressure to 36.8 cm H_2_O.

#### 6.1.2. Enterocystoplasty

Enterocystoplasty, in which bladder capacity is increased (and its pressure simultaneously lowered) by anastomosing a part of the ilium or ileocecal segment to the detrusor, is generally accepted as the most effective surgical method to achieve an adequate reservoir of urine. The success rate of enterocystoplasty is reported to be as high as 90% [59]. Bladder augmentation has been performed in 25–30% of individuals with meningomyelocele [32]. This procedure is major surgery, however, with an overall complication rate of up to 40% in the series with the longest mean followup [60]. Surgical complications of enterocystoplasty include infections, anastomotic leakage, strictures, adhesions, intestinal obstruction, urinary fistulas, enteric fistulas, and bladder rupture [59]. Robotic enterocystoplasty has been described [61] which may reduce some of the surgical complications of this procedure. Other potential complications of enterocystoplasty include mucus formation, bladder calculi (2–18%), electrolyte disturbances, and an elevated risk of bladder adenocarcinoma (approximately 1%).

After enterocystoplasty, the neurogenic bladder patient may be able to void normally, but it is possible that an external urine collection device may be required. The number of patients needing to perform intermittent catheterization following enterocystoplasty has been reported to range from 15%–75% [59].

Bladder autoaugmentation by creating a large detrusor diverticulum via myomyotomy or myomectomy has also been performed to increase bladder capacity and reduce intravesicular pressure. Operative results often were not optimal in the past due to a bladder diverticulum either failing to form or being of inadequate capacity. Postoperative ischemia and fibrosis of the diverticulum as well as recession of the mucosa have been postulated as potential mechanisms leading to inadequate diverticulum formation. More recently, the use of an inflatable silicon balloon placed in the bladder after autoaugmentation has been described [62] which has been reported to improve the success rate of this procedure to 80% with a 190–380% increase of bladder capacity and normal bladder compliance [63, 64].

Experimentally, tissue engineering techniques for bladder augmentation are being studied including unseeded or seeded biodegradable biomatrices. Though animal studies demonstrate that urothelial regeneration can occur with a variety of graft materials, muscular layer regeneration did not occur in synthetic or acellular grafts resulting in fibrosis and contraction of the graft in 60–70% of cases [65–67]. Implants made from collagen or PLGA-based scaffolds seeded with autologous progenitor cells have had preliminary positive results in small clinical studies [68].

### 6.2. Procedures to Control Detrusor Emptying

#### 6.2.1. Urinary Diversion

For patients who have a neurogenic bladder with incomplete emptying (lower motor neuron bladder, mixed type A neurogenic bladder, or following bladder augmentation procedures) and who have disabilities that make self-catheterization through the urethra impractical, a continent abdominal stoma can be created for clean intermittent catheterization surgically with a >90% success rate. It still requires a major abdominal surgery, however.

Appendicovesicostomy anastomosis can be performed on the anterior detrusor wall without the need for bladder augmentation, or it can be performed on the posterior bladder wall in conjunction with augmentation. The stoma can be brought to the umbilical area or to the right lower quadrant. By having at least 4 cm of detrusor backing, stomal continence can be maintained. Pedicled skin flaps have also been utilized to form catheterizable channels [69, 70].

#### 6.2.2. Bladder Sphincter Procedures to Enhance Emptying

**Sphincterotomy**

Sphincterotomy, first described by Ross et al. in 1956 [71], has largely been supplanted by use of botulinum toxin injections, medications, or urethral stents. By reducing the urinary outlet obstruction, reflex voiding occurs with less detrusor pressure and improved bladder emptying. Fewer urinary tract infections and reduced risk of autonomic dysreflexia are additional benefits. Most patients undergoing sphincterotomy have resulting incontinence due to urinary leakage, so this procedure is generally not an appropriate choice for women or if use of a condom catheter is not plausible due to body habitus in male patients. Indications for sphincterotomy include detrusor sphincter dyssynergia with hydronephrosis, vesicoureteral reflux, and autonomic dysreflexia or recurrent urinary tract infections due to poor bladder emptying [58]. This procedure is likely most appropriately reserved for quadriplegic males who are unable to perform self-intermittent catheterization. Up to 50% of patients with detrusor areflexia can have successful results with sphincterotomy.

Hemorrhage is a significant complication of sphincterotomy, but postoperative bleeding may be reduced by using laser rather than a knife to create the sphincter sectioning [58]. Erectile dysfunction may occur in 3–7% of males undergoing sphincterotomy, and this procedure results in bladder neck or urethral strictures in 3–13% patients that will require reoperation [58]. Three-quarters or more of patients undergoing this procedure will have resolution of vesicoureteral reflux and recurrent urinary tract infections, and over 90% will experience resolution of autonomic dysreflexia. Despite these benefits of sphincterotomy, there is still an approximate 30% chance that these persons will undergo upper urinary tract deterioration over time. A leak point pressure (LPP) of over 40 cm H_2_O on urodynamic study after sphincterotomy is associated with a significantly higher incidence of upper tract damage and persistent detrusor sphincter dyssynergia [58]. Persistent obstruction due to bladder neck obstruction can be managed by a trial of alpha adrenergic blocking drugs, bladder neck incision/resection, or use of an indwelling urinary catheter (urethral or suprapubic).

**Urethral Stents and Balloon Dilatation**

Urethral stents can serve as a therapeutic alternative to sphincterotomy both as a primary procedure or to treat a failed sphincterotomy procedure [72]. By strict avoidance of putting the stent through the bladder neck, the incidence of stent encrustation and development of dysreflexia can be minimized. Though stent migration may occur, the stents usually can be repositioned or removed with low incidence of significant urethral trauma. Recurrent urinary tract infections may be a relative contraindication to this procedure [73] due to risk of stent infection. This procedure may have a theoretical advantage over sphincterotomy in that weight gain, wheelchair seating position, and pelvic floor spasticity may well cause functional urethral obstruction that will not be detected by urodynamic studies.

Balloon dilatation of the external sphincter has also been studied and produces outcomes at 12 months that are comparable to sphincterotomy [74] Long-term followup, however, suggests that the results of balloon dilatation of the external urethral sphincter may not produce durable results [75].

#### 6.2.3. Bladder Sphincter Procedures to Restrict Emptying

**Artificial Urinary Sphincter**

Artificial urinary sphincter devices are presently the “gold standard” method to treat urinary incontinence due to internal and/or external urinary sphincter incontinence, with a 75–95% social continence rate (<1 incontinence pad use per day) reported at three year followup in male, female, and pediatric populations [76].

Implantable artificial urinary sphincters have been in clinical use since 1972, but early devices had high mechanical failure and urethral erosion rates. Over the next decade, design improvements produced more reliable devices that produced acceptable complication rates; and nearly 100,000 artificial urinary sphincters have been implanted over the last three decades. This intervention may be considered in neurogenic bladder patients with adequate bladder capacity and compliance, who have internal and/or external urinary sphincter incompetence resulting in urinary incontinence unresponsive to bladder retraining and pharmacologic approaches. Further, these patients must have sufficient learning capacity and hand strength/dexterity to operate the pump and its valve mechanisms.

If bladder capacity and/or compliance are low, bladder augmentation procedures may be combined with artificial sphincter replacement for treatment of the neurogenic bladder dysfunction. Urethral diverticula or strictures are contraindications to the artificial urinary sphincter. Relative contraindications include recurrent bladder stones, ≥ grade 2 vesicoureteral reflux (which may worsen after the artificial urinary sphincter placement), or urethral or bladder tumors that will require future repeated urethral instrumentation (repeated instrumentation can damage the artificial sphincter device's urethral cuff).

Up to 35% of patients undergoing artificial sphincter implantation may require reoperation, with about half of these reoperations due to device mechanical failures. Loss of fluid from the artificial sphincter hydraulic system producing loss of cuff filling and hence recurrent incontinence is the most common reason for device failure but air in the system, debris, blood, and/or crystals may obstruct urinary flow. The artificial urinary sphincter devices generally last for approximately a decade. The most common nonmechanical reason for reoperation is urethral tissue atrophy leading to inadequate urethral compression from the cuff resulting in recurrent urinary leakage (3–9%). Deflation of the artificial urinary sphincter cuff overnight may reduce the risk of urethral tissue atrophy. Replacement of the cuff with a smaller cuff, tandem cuff placement [77], and higher balloon pressure (to increase urethral closing pressure) are treatment options for artificial urinary sphincter failure; due to tissue atrophy. Device infections (2-3%) may occur, and cuff erosions may occur in 1–3% with early erosions (<4 months postoperativly) thought likely to relate to unrecognized bladder neck or urethra trauma, and late erosions may be due to intermittent catheterization, cuff infection, or pressure necrosis from excessive balloon pressure or undersized cuff [78, 79]. Retrograde instrumentation such as cystoscopy or catheterization, and prior pelvic radiation are also risk factors for cuff erosions. If cuff erosion into the urethra occurs, a catheter is placed for 3-4 weeks to allow the urethra to heal. Typically, surgery to place a new cuff would occur 3–6 months later. Rarely, cuff migration may occur.

**Sling Procedures**

Artificial urinary sphincter and/or bladder neck reconstruction is commonly used clinically to treat neurogenic detrusor overactivity with low Valsalva leak point pressure (as is often seen with spinal cord injury with damage to thoracolumbar sympathetic outflow). Rates of artificial urinary sphincter device infection and erosion, however, are higher in persons with neurogenic bladder due to this type of cord injury [80]. Thus, a bulbourethral sling procedure in conjunction with a bladder augmentation procedure may be a reasonable alternative in this clinical setting. If detrusor contractility is impaired such as in lower motor neuron or mixed type B neurogenic bladder, a bulbourethral sling procedure may lead to urinary retention. An artificial urinary sphincter may be better option if catheter free voiding is the goal of surgical treatment.

A 66–83% success rate for the bulbourethral sling procedure is reported with rare complications after one-year followup [81, 82], but the long-term efficacy of this procedure is unknown.

## 7. Future Developments

### 7.1. Lumbar to Sacral Nerve Rerouting

Restoring bladder function in spina bifida by creation of a skin-central nervous system bladder reflex arc via lumbar (L5 motor) to sacral (S3) nerve rerouting has a reported success rate of 87% in 110 children in China [83]. Recently the one-year results of the first North American trial were reported, with 7/9 (87%) of spina bifida subjects having measurable increase in bladder pressure with L5 dermatomal stimulation (>10 cm H_2_O), most demonstrating a modest increase in bladder compliance, and all stopping antimuscarinic drugs. Two subjects were able to stop catheterization, but none achieved complete urinary continence [84]. One patient had a persistent foot drop after this surgery. These outcomes differ substantially from the Chinese experience, and the improvements in continence and bladder compliance may relate to sectioning of the S3 nerve root in the procedure. This should still be considered an experimental procedure until further prospective data on its efficacy and effects on quality of life can be determined.

### 7.2. Spinal Cord Regeneration

Transplantation of fibroblasts genetically modified to express brain-derived neurotrophic factor (BDNF) and neurotrophin 3 (NT-3) or transplantation of neuronal and glial precursors into the spinal cord lesions experimentally induced in rats has been reported to result in decreased detrusor pressures and less frequent detrusor hyperreflexia episodes [85, 86]. Implantation of neurotrophin-secreting Schwann cells into rat spinal cord lesions one hour after induced thoracic cord injury led to improved bladder morphology and bladder capacity [87]. Park et al. [88], however, noted that implantation of mesenchymal stem cells 9 days after experimental thoracic cord injury in rats did not significantly improve bladder or hindlimb function and noted no increase in BDNF or NT-3 levels in the bladder or spinal cord after 28 and 56 days.

Olfactory ensheathing cells (OECs), which are glial cells derived from the olfactory placode, have axonal growth promoting properties in their interaction with astrocytes and thus have been studied to see if they can promote spinal cord injury repair. In a pilot study of 30 human subjects with chronic paraplegia or tetraplegia, Lima et al. [89] transplanted olfactory mucosal cell autografts into the area of prior cord damage with at least one-year (and average of over 2 year) followup. American Spinal Cord Injury Association scores improved in 11 subjects but declined in one, and urodynamic responses were reported improved in 5 subjects.

In spinal cord injury, reactive astrocytes form glial scar at the site of injury and secrete growth inhibiting chondroitin sulfate proteoglycans. Epidermal growth factor (EGF) may stimulate astrocytes to become reactive astrocytes in these areas of injury, so pharmacologic blockade of EGF receptors on astrocytes in the site of injury may mitigate damage there and enhance neurologic recovery (including bladder function). Erschbamer et al. [90] chronically blockaded EGF receptors in an acute spinal cord injury in rats using an osmotic pump subdural infusion of PD168393, an EGF receptor inhibitor, for 21 days over the site of cord injury. Locomotor function was found to be improved in the treated rats, and bladder function was reported as improved as measured by residual urine volume (though the absolute difference was only about 2 cc residual urine volume per day). Chondroitinase ABC intrathecally has also been tested alone or in combination with cellular transplantation of OECs and Schwann cells in rats and has been reported to improve recovery of bladder function in experimentally spinal cord injured rats at short-term followup [91].

## 8. Conclusions

Neurogenic bladder dysfunction can be successfully treated to achieve goals of urinary continence, prevention of renal damage from chronically high detrusor pressures, and minimizing risk of urinary tract infections or bladder overdistension. A comprehensive multidisciplinary bladder retraining program can best achieve these goals utilizing patient education, instruction in catheter use/care, medications, and/or bladder or urethral surgical procedures. Experimental works in lumbar-to-sacral nerve rerouting and in regenerative medicine including use of stem cells to mitigate or reverse spinal cord damage producing neurogenic bladder dysfunction are still in their infancy, and more research will be needed to see if the promising results of some small pilot studies are confirmed in larger, controlled studies with long-term followup.
